# Brain morphometry and diminished physical growth in Bangladeshi children growing up in extreme poverty: A longitudinal study

**DOI:** 10.1016/j.dcn.2021.101029

**Published:** 2021-10-26

**Authors:** Ted K. Turesky, Talat Shama, Shahria Hafiz Kakon, Rashidul Haque, Nazrul Islam, Amala Someshwar, Borjan Gagoski, William A. Petri, Charles A. Nelson, Nadine Gaab

**Affiliations:** a Laboratories of Cognitive Neuroscience, Division of Developmental Medicine, Department of Medicine, Boston Children’s Hospital, Boston, MA, United States; b Harvard Graduate School of Education, Cambridge, MA, United States; c Harvard Medical School, Boston, MA, United States; d The International Centre for Diarrhoeal Disease Research, Dhaka, Bangladesh; e National Institute of Neuroscience and Hospital, Dhaka, Bangladesh; f Fetal-Neonatal Neuroimaging and Development Science Center, Boston Children’s Hospital, Boston, MA, United States; g Division of Infectious Diseases and International Health, Department of Medicine, School of Medicine, University of Virginia, Charlottesville, VA, United States

**Keywords:** Adversity, Brain, Morphometry, MRI, Poverty, Stunting

## Abstract

Diminished physical growth is a common marker of malnutrition and it affects approximately 200 million children worldwide. Despite its importance and prevalence, it is not clear whether diminished growth relates to brain development and general cognitive ability. Further, diminished growth is more common in areas of extreme poverty, raising the possibility that it may mediate previously shown links between socioeconomic status (SES) and brain structure. To address these gaps, 79 children growing up in an extremely poor, urban area of Bangladesh underwent MRI at age six years. Structural brain images were submitted to Mindboggle software, a Docker-compliant and high-reproducibility tool for tissue segmentation and regional estimations of volume, surface area, cortical thickness, sulcal depth, and mean curvature. Diminished growth predicted brain morphometry and mediated the link between SES and brain morphometry most consistently for subcortical and white matter subcortical volumes. Meanwhile, brain volume in left pallidum and right ventral diencephalon mediated the relationship between diminished growth and full-scale IQ. These findings offer malnutrition as one possible pathway through which SES affects brain development and general cognitive ability in areas of extreme poverty.

## 1 Introduction

Early adverse experiences can substantially derail typical child development (Nelson and Gabard-durnam, 2020). This derailment is especially pronounced in communities of extreme poverty, where biological hazards such as malnutrition prevent children from reaching their full growth potential (Grantham-McGregor et al., 2007, John et al., 2017), instead causing diminished growth (Bhutta et al., 2017a, Black et al., 2013, de Onis and Branca, 2016, Schnee et al., 2018, Stewart et al., 2013), poor neurocognitive outcomes (Fuglestad et al., 2008, de Onis and Branca, 2016, Xie et al., 2019a), and premature death (Black et al., 2013, Hayashi et al., 2018, de Onis and Branca, 2016).

Globally, over 300 million children grow up in extreme poverty (UNICEF; https://www.unicef.org/social-policy/child-poverty). To monitor the impact of biological and psychosocial hazards on these children, many low-resource countries use forms of diminished growth quantified by the World Health Organization anthropometric indicators. Namely, stunting is measured with height-for-age (HAZ); underweight is measured with weight-for-age (WAZ); and wasting is measured with weight-for-height (WHZ) (de Onis et al., 2006, de Onis et al., 2012). Stunting, is the most common form of diminished growth, occurring worldwide in over 150 million children under five years of age (Hayashi et al., 2018), over 60 million of whom are from South Asia (Black et al., 2017). These measures are thought to reflect a combination of environmental factors, including malnutrition, infection, and psychosocial care or deprivation (Nahar et al., 2009, Stewart et al., 2013, de Onis and Branca, 2016). Although its multifactorial nature limits the precision with which constituent factors can be measured, diminished growth is commonly employed as a proxy for malnutrition (Black et al., 2013, de Onis and Branca, 2016, Schnee et al., 2018), with HAZ more reflective of chronic childhood malnutrition and WHZ more related to severe or moderate acute malnutrition due to protein deficiency (Bhutta et al., 2017a, Galler et al., 2021).

Diminished growth is also associated with reduced cognitive outcomes (Fuglestad et al., 2008, de Onis and Branca, 2016, Donowitz et al., 2018, Xie et al., 2019a), leading to the hypothesis that it would also be associated with altered brain structure (Jensen et al., 2017). Indeed, greater stunting has been associated with less total white matter volume in infancy (Turesky et al., 2020). These findings bolster earlier work describing cerebral atrophy in young children exhibiting wasting and acute malnutrition (Gunston et al., 1992, Hazin et al., 2007, Atalabi et al., 2010, El-Sherif et al., 2012, Kumar et al., 2016; c.f., Lelijveld et al., 2019), reduced brain volume in adolescents and young adults with lower prenatal nutritional markers (Ivanovic et al., 2004), and decreased intracranial volume in adults exposed to malnutrition prenatally (Hulshoff et al., 2000). White matter atypicalities (e.g., atrophy) in children exhibiting wasting were also observed in all reports, but to highly varying degrees depending on the type of atypicality examined (e.g., atrophy or delayed myelination; Gunston et al., 1992; Hazin et al., 2007; Atalabi et al., 2010; El-Sherif et al., 2012; Kumar et al., 2016). Overall, few neuroimaging studies have examined children exposed to significant malnutrition in settings of extreme poverty and these have been limited to gross, qualitative characterizations of brain structure (e.g., presence or absence of cerebral atrophy), rather than comprehensive, quantitative estimations of brain morphometry. Further, these prior studies have focused on children experiencing wasting and acute malnutrition, rather than on the chronic malnutrition reflected by stunting.

A further consideration when examining malnutrition is the role of poverty. In high-resource settings, socioeconomic status (SES) has been linked with total brain volume, total gray and white matter volumes, total surface area, and average cortical thickness (McDermott et al., 2019). Regional hippocampus (Hanson et al., 2011, Noble et al., 2012, Noble et al., 2015, Luby et al., 2013, McDermott et al., 2019), amygdala (Noble et al., 2012, Luby et al., 2013, McDermott et al., 2019), thalamus (McDermott et al., 2019), and striatum (McDermott et al., 2019) volumes were also associated with SES. However, it is unclear whether the brain measures and regions associated with SES in high-resource countries would persist in settings of extreme poverty, defined by the World Bank as $1.90 per person per day (https://data.worldbank.org/).

A substantial corpus of literature has been devoted to understanding the mechanisms through which SES disadvantage may alter brain development (Raizada and Kishiyama, 2010, Gianaros and Hackman, 2013, Johnson et al., 2016, Jensen et al., 2017, Lipina and Evers, 2017, Farah, 2018). In doing so, this literature has mainly drawn on studies examining how various psychosocial risk factors directly act on the brain. Broadly speaking, much of the focus has been allocated to the study of stress, including its conceptualization (Mclaughlin et al., 2014a, Sheridan and McLaughlin, 2014, Blair and Raver, 2016, Tooley et al., 2021), its neural correlates (Hanson et al., 2015, Piccolo and Noble, 2018, Tyborowska et al., 2018), and the neuronal and endocrine processes it affects (McEwen and Gianaros, 2010, McEwen et al., 2016). There is a growing consensus that many stressful experiences can be subdivided into deprivation (e.g., institutionalization, which deprives children of an expected caregiver relationship) and threat (e.g., familial conflict) (Sheridan and McLaughlin, 2014), both of which have been associated with alterations in early brain structure (Rao et al., 2010, Tottenham et al., 2010, Sheridan et al., 2012, McLaughlin et al., 2014b, Lebel et al., 2016, Wen et al., 2017, Spann et al., 2020, VanTieghem et al., 2021). Lack of expected linguistic stimulation can represent another form of psychosocial deprivation, but it may also constitute a separate risk factor (Merz et al., 2020), as low SES can mean insufficient funds for reading and other educational material (Johnson et al., 2016). Though rarer in studies of human development, biological risk factors have also been investigated. For instance, inflammation, which is a common concern in low-resource settings (Bhutta et al., 2017b, John et al., 2017, Kutlesic et al., 2017), has been associated with white matter atypicalities (Yoon et al., 1997, Duggan et al., 2001, Hansen-Pupp et al., 2005) and amygdala volume (Graham et al., 2018). Additionally, reduced white matter volume has been attributed to heightened air pollution (Calderón-Garcidueñas et al., 2011). In part due to the dearth of neuroimaging facilities in low-resource settings where these biological risk factors are more prevalent (Gianaros and Hackman, 2013, Galler et al., 2021), there is a salient gap between the examination of psychosocial and biological hazards in the context of brain development.

Further, in spite of the various mechanisms that have been examined to explain associations between SES and brain development, few studies have directly tested potential risk factors in formal mediation analyses, which are necessary (though not sufficient) to establish causal pathways (Farah, 2017). An early exception to this was a study by Luby and colleagues, which observed indirect effects between SES and hippocampal volume via stressful life events and caregiving quality (Luby et al., 2013). Bolstering this, a more recent report found that the concentration of cortisol, the canonical stress hormone, mediated the relation between SES and CA3 and dentate gyrus (hippocampus) volumes (Merz et al., 2019). The same group later found that linguistic input at home mediated the association between SES and left perisylvian cortical surface area (Merz et al., 2020). To our knowledge, malnutrition and diminished growth have not been statistically tested as mediators in the link between SES and brain development, which represents a critical gap in the literature examining the SES-brain relationship. Understanding these mechanisms will be important for prevention or intervention programs designed to remedy deleterious consequences of SES disadvantage (Olson et al., 2021).

**Fig. 1:**
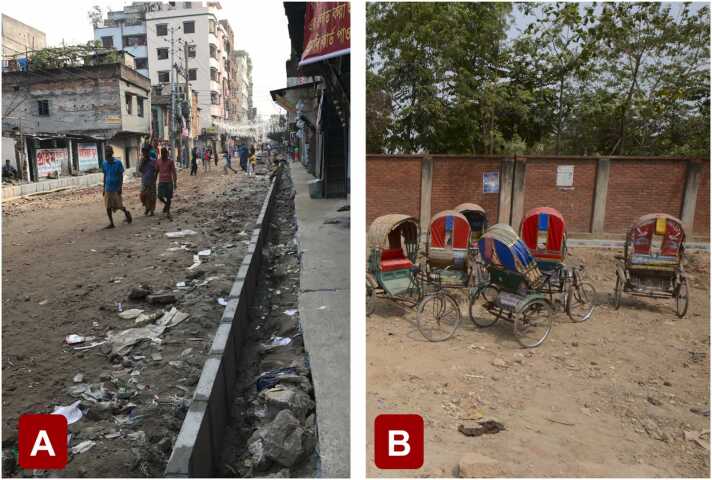
Study setting. (A) Open sewars and insufficient waste disposal in public spaces and (B) rickshaws for transportation.

One reason this gap has persisted is that very few studies have examined children in a part of the world where diminished growth is prevalent, namely South Asia, which claims the greatest percentage (40%) of children under five years of age who experience stunting (Black et al., 2017). Children growing up in urban areas of Bangladesh are particularly vulnerable to diminished growth, as many who reside in these regions experience severe malnutrition due to food scarcity and chronic enteric disease, as well as a broad spectrum of other biological and psychosocial hazards (Nelson, 2017). The overcrowding and unsanitary conditions exacted by the urban environment (e.g., open sewars, insufficient waste disposal; Fig. 1A) may exacerbate these adversities. Overall, the type and severity of poverty in these environments is unparalleled in high-resource settings (e.g., the United States).

The present study addresses three main lines of inquiry. The first line is whether diminished growth, a proxy for malnutrition (Black et al., 2013, de Onis and Branca, 2016, Schnee et al., 2018, Stewart et al., 2013), predicts subsequent brain structure. Diminished growth has been linked to measures of brain morphometry in infants (Turesky et al., 2020), but it is not clear how these variables relate in children who have lived in extreme poverty for a longer duration (e.g., several years). To address this, we collected anthropometric measures at approximately two and a half years of age and structural MRI at six years of age in 79 children growing up in an extremely poor, urban area of Bangladesh, a vastly underrepresented population in neuroscientific research.

To comprehensively quantify global and regional brain morphometry at a level of detail not reached by past neuroimaging reports on diminished growth or malnutrition, MRI data were submitted to Mindboggle (Klein et al., 2017), a software that estimates volume, surface area, cortical thickness, sulcal depth, and mean curvature in a Docker container for high reproducibility (Gorgolewski et al., 2017). Volume relates to both surface area and cortical thickness, but the latter are phenotypically and genetically independent of each other (Winkler et al., 2010), suggesting they offer distinct characterizations of brain structure. We hypothesized associations between diminished growth and brain volume, surface area, and cortical thickness based on common links between these measures and SES (Grantham-McGregor et al., 2007, McDermott et al., 2019). Further, we expected associations with volume to include white matter, as links between anthropometric measures and total white matter volume have been observed previously in infants (Turesky et al., 2020) and white matter atypicalities have been observed in children exhibiting severe wasting (Gunston et al., 1992, Hazin et al., 2007, Atalabi et al., 2010, El-Sherif et al., 2012, Kumar et al., 2016). However, our approach to regional effects was exploratory, as the literature linking anthropometric measures and malnutrition to brain structure did not provide a strong foundation for limiting analyses to particular brain regions a priori.

Lesser-utilized brain morphometric measures were also examined. Sulcal depth measures the distance between points on the cortical surface and an outer reference surface that expands across gyri without dipping into sulci, and mean curvature measures local folding of sulci and gyri (Van Essen, 2005, Klein et al., 2017). There is no evidence yet that these lesser-utilized surface-based measures relate to diminished growth or SES. However, sulcal depth has been shown to be sensitive to early developmental atypicalities (Shimony et al., 2016), suggesting that it may also relate to diminished growth. In contrast, mean curvature exhibited no such sensitivity (Shimony et al., 2016) and by comparison with other morphometric measures, has been shown to change exceedingly little (average 1%) across early development (Remer et al., 2017), suggesting it is unlikely to relate to environmental factors such as those reflected by diminished growth.

The second line of inquiry concerns the broader context of SES in altering brain structure. Particularly, we hypothesized that SES would relate to brain morphometry in low-resource settings as it did in high-resource settings (Noble et al., 2012, Noble et al., 2015, Mackey et al., 2015, McDermott et al., 2019). SES was measured when children were just under two years old using two factors commonly examined in developmental cognitive neuroscience studies of SES in high-resource settings: maternal education and income-to-needs (the ratio of household income to number of household members) (Hanson et al., 2011, Noble et al., 2012, Noble et al., 2015, Brito and Noble, 2014). We also expected that diminished growth would mediate these associations based on its relation to SES in three year-old children (Jensen et al., 2019a). The third line of inquiry addresses whether brain morphometry mediates associations between diminished growth and general cognitive ability.

## 2 Materials and methods

### 2.1 Participants

The present study is part of the Bangladesh Early Adversity Neuroimaging study (Jensen et al., 2019a, Jensen et al., 2019b, Moreau et al., 2019, Turesky et al., 2019, Turesky et al., 2020, Xie et al., 2019a, Xie et al., 2019b), investigating effects of extreme poverty on early brain development in children in Dhaka, Bangladesh. The overall study followed 260 children beginning in infancy, from whom socioeconomic, anthropometric, behavioral, and biological measures were collected. The current study focused on the 81 children who underwent structural MRI between ages six and eight years. After excluding children with severe motion artifacts, the final sample comprised 79 structural MRI datasets (please see Table 1 for demographic details). No child had been diagnosed with a neurological disease or disability and all scans were reviewed for malignant brain features by a clinical radiologist in Bangladesh and a pediatric neuroradiologist at Boston Children’s Hospital. The study was approved by the research and ethics review committees at BCH and The International Centre for Diarrhoeal Disease Research, Bangladesh.

**Table 1:** Participant demographics.

N	79
Sex (F/M)	36/43
Age at MRI (Years)	6.7±0.40
Age range at MRI (Years)	5.5–7.0
HAZ^a^	-1.6±0.89
WAZ^a^	-1.3±1.1
WHZ^a^	-0.66±1.1
Maternal education (Years)^b^	4.4±3.9
Income-to-needs^c^	3100±1700
Full-scale IQ	86±9.1

a Scores less than −2 indicate stunting, underweight, or wasting (Grantham-McGregor et al., 2007).

b Rescaled (please see Methods).

c Monthly household income in Bangladeshi Taka/number of household members.

### 2.2 Anthropometric measures

Height-for-age (HAZ), weight-for-age (WAZ), and weight-for-height (WHZ) scores were used to estimate stunting, underweight, and wasting, respectively. Trained, local staff measured height (in centimeters) and weight (in kilograms). These measures were then submitted to Anthro Plus software (https://www.who.int/tools/child-growth-standards/software; Multicenter Growth Reference Study de Onis et al., 2004) and compared with growth curve data from 8440 infants (0–24 months) and children (18–71 months) from Brazil, Ghana, India, Norway, Oman and the U.S. Resulting z-scores (i.e., HAZ, WAZ, and WHZ), which were age- and sex-referenced and standardized, reflected deviations from typical growth trajectories. Critically, the standard growth curves comprised infants and children who grew up in healthy environments (including with breastfeeding and absence of smoking) that are “likely to favour the achievement of their full genetic growth potential” (de Onis et al., 2006). As such, consistent deviations from these standard growth curves can be inferred to reflect environmental hazards during upbringing. Lastly, anthropometric measures were collected at 21, 30, and 36 months and these values were averaged to ensure stability. There were robust intercorrelations among measures acquired at 21, 30, and 36 months for HAZ (r_avg_ = 0.93, p_avg_ = 2.8 × 10^-^^32^), WAZ (r_avg_ = 0.94, p_avg_ = 1.9 × 10^-32^), and WHZ (r_avg_ = 0.88, p_avg_ = 2.0 × 10^-22^). Stunting, underweight, and wasting were respectively defined as HAZ, WAZ, and WHZ less than −2 (Grantham-McGregor et al. 2007). According to this definition, 24 children were stunted, 18 children were underweight, and 8 children were wasted. Six children were stunted, underweight, and wasted. Table 1 summarizes these measures in the final cohort.

### 2.3 Measures of socioeconomic status (SES)

Years of maternal education, monthly household income, and number of household members were used to compute two measures of SES: maternal education and income-to-needs ratio. Examining both maternal education and income-to-needs, separately and along a gradient or continuous scale, fosters a more thorough understanding of how SES affects development and is consistent with approaches currently recommended in behavioral literature (Adler et al., 1994, Duncan et al., 2017) and implemented in brain imaging literature (Lawson et al., 2013, Noble et al., 2015, Betancourt et al., 2016, Brito et al., 2016, Merz et al., 2018). Maternal education was measured as years of formal education, ranging from 0 to 10, with 0 indicating no formal education, 1–9 indicating number of grades passed, and 10 indicating education beyond the 9th grade passed, in which degrees may be conferred. Income-to-needs was computed as the monthly family income divided by the number of household members. As the exchange rate for USD to Bangladeshi taka is USD$1:Tk85, the family in the cohort with the lowest monthly income-to-needs earned Tk890 or USD$10 per household member per month, while the family in the cohort with the highest monthly income-to-needs earned Tk10,000 or USD$120 per household member per month. When considering that the World Bank (https://data.worldbank.org/) international standard for extreme poverty is roughly Tk4800 or USD$57 per person per month (calculated from USD$1.90/day and assuming 30 days/month), the final cohort constituted 71 out of 79 children living in extreme poverty. When considering the national poverty line of Tk2242 or USD$26/person/month, 30 out of 79 children live in poverty (bbs.gov.bd). Maternal education and income-to-needs were assessed twice—once at age six months and a second time at age 36 months—through oral interviews with the children’s parents, and then averaged, to better capture overall measures of SES that reflected the entire childhood. There were robust correlations between measures acquired at 6 and 36 months for maternal education (r = 0.94, p = 2.4 × 10^-37^) and income-to-needs (r = 0.67, p = 8.7 × 10^-12^). Due to a positive skew in the income-to-needs variable, these data were log (base 10) transformed.

### 2.4 General cognitive ability

Children underwent general cognitive testing using the Wechsler Preschool and Primary Scale of Intelligence (WPPSI) administered by trained, local psychologists, and staff. Although the WPPSI has been standardized using U.S. children, steps were taken to ensure translatability in this Bangladeshi cohort. For instance, items in the assessment were translated and culturally adapted (Jensen et al., 2019a) and the Bangladeshi version of the assessment exhibited test-retest reliability (Hamadani et al., 2011, Kippler et al., 2012). The full-scale intelligence quotient (FIQ) score, which reflects children’s general cognitive abilities, was tested for associations with anthropometric and brain morphometric measures. While WPPSI was assessed at age five years, prior to MRI scanning (conducted between ages 5.5 and 7.0 years), these tests are considered relatively stable after four years of age (Sameroff et al., 1993).

### 2.5 MRI data acquisition

Neuroimaging data were acquired on a 3T Siemens MAGNETOM Verio scanner using a 12-channel head coil at the National Institute for Neuroscience and Hospital (NINSH) in Dhaka, Bangladesh. Consenting was done at NINSH on the day prior to scanning. For consenting and scanning, children and their mothers were brought to and from NINSH by study staff, usually via rickshaw (Fig. 1B). The cost of transport was paid for by the study, and children and mothers were provided meals on the day of the scan.

NINSH previously scanned pediatric patients for clinical examinations and for pilot studies in infants (Turesky et al., 2019, Turesky et al., 2020), but this study marks the first time this facility had collected MRI data for a large-scale pediatric neuroimaging study. As such, local staff visited Boston Children's Hospital to receive training on conducting pediatric MRI studies (Raschle et al., 2009). Subsequently, a protocol was designed that incorporated this training and the limitations of a low-resource setting (for a general description of challenges of conducting MRI in a low resource setting, please see Turesky et al., 2019). Specifically, children were brought to NINSH by trained, local staff. Prior to scanning, children were shown sample anatomical images of the brain to teach them about the purpose of their visit and to explain that the machine they would enter would take pictures of their brains. They then went through several steps to practice remaining motionless. First, they were shown MRI images from a child remaining motionless during scanning (i.e., a clear image) and a child moving during scanning (i.e., a blurry image) to understand the effects of motion. Second, staff played a "freeze tag" game with them, such that children practiced becoming still when staff instructed them to "freeze." Third, they were asked to lie motionless for 1 min and offered encouragement for doing so. If they moved while doing this, they were told that any tapping they felt on their feet meant that they need to remain still.

**Fig. 2:**
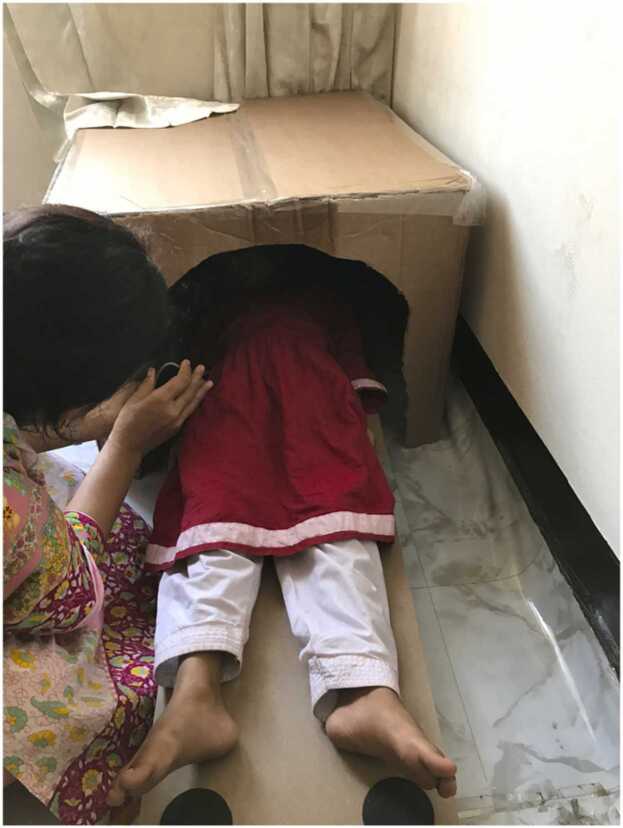
Cardboard mock scanner at MRI facility.

Following this training, children entered a cardboard mock scanner to familiarize them with the scanning environment (Fig. 2). Staff narrated throughout to ensure the children would not be scared. Children again received a tapping on their feet to practice lying motionless. To ensure children would become familiar with scanner noise, staff tapped on the outside of the cardboard box and played MRI sounds previously recorded on a CD. Children were then shown pictures of the real scanner so that they could connect its makeup to that of the mock scanner.

Structural T1-weighted magnetization-prepared rapid gradient-echo (MPRAGE) scans were acquired with the following parameters: TR = 2500 ms, TE = 3.47 ms, 176 sagittal slices, 1 mm^3^ voxels, FOV = 256 mm. Functional and diffusion scans were also acquired but these modalities will be described in future reports. Head circumference was also measured at the time of scan. All demographic, anthropometric, SES, cognitive, and neuroimaging data used in main analyses have been made openly available at https://openneuro.org/datasets/ds003877/versions/1.0.1.

### 2.6 MRI data processing

Images were visually inspected for artifacts. Following the removal of two artifactual datasets due to head motion, the remaining raw MPRAGE images were processed using Mindboggle 1.3.8, run in a Docker container (https://Mindboggle.readthedocs.io/en/latest/; Klein et al., 2017). This pipeline implements Advanced Normalization Tools (ANTs) and FreeSurfer (v6.0.0). First, Mindboggle calls antsCorticalThickness, which includes brain extraction, N4 bias correction, tissue segmentation, and cortical thickness estimation. Subsequently, Mindboggle submits raw images to recon-all, which segments the brain into different tissue classes, approximates pial surfaces, and labels volumes and surfaces by brain region.

The final series of steps belong to Mindboggle proper. First, FreeSurfer output is converted to nifti and vtk filetypes for combining with ANTs. Second, Mindboggle runs a hybrid segmentation algorithm to reconcile differences between ANTs and FreeSurfer segmentations. These toolkits separately mislabel tissue classes in different ways, with ANTs underestimating white matter and including non-brain tissue, while FreeSurfer omits gray matter voxels by overcropping. Third, volumetric measures are computed for cortical and subcortical brain regions (including white matter), while surface area, cortical thickness (from FreeSurfer), sulcal depth, and mean curvature measures are computed for each cortical brain region. Two sets of measures were provided for brain volume of various structures—one using ANTs labels and another using FreeSurfer labels. We opted to use the latter for two reasons. First, cortical thickness measures were reported as FreeSurfer-derived. Second, FreeSurfer’s labels were more comprehensive for white matter by comparison with ANTs and white matter has been particularly relevant in the context of diminished growth (Turesky et al., 2020). Mindboggle also offers two measures of sulcal depth: travel and geodesic. We limited our analyses to travel depth; however, these two measures are highly similar across the brain except in insular regions (Klein et al., 2017), where we did not have specific hypotheses. Laplace-Beltrami spectrum and Zernicke moments output by Mindboggle were also excluded from analyses. As with other neuroimaging tools that run via the Docker (e.g., fMRIPrep; Esteban et al., 2019), this pipeline is highly reproducible.

After processing, we examined global measures of brain volume (total brain volume, total gray matter volume, total white matter volume) and global surface-based measures (total surface area, average cortical thickness, average sulcal depth, and average mean curvature). Mindboggle outputs these brain measures by region, making computations of global estimates for volume and surface area straightforward via summing, but hampering global estimations for cortical thickness, sulcal depth, and mean curvature, for which summing would have been difficult to interpret due to variation in region size. To circumnavigate this challenge, we computed average cortical thickness, average sulcal depth, and average mean curvature weighted by surface area in each region. We then examined regional measures of brain volume from cortical, subcortical, and white matter areas, as well as regional surface-based measures (surface area, cortical thickness, sulcal depth, and curvature). Lastly, one volumetric estimate was by default labelled as ‘unsegmented white matter.’ From visual inspection, we observed that voxels classified as ‘unsegmented white matter’ were predominantly localized to corona radiata and internal capsule, but these two fiber pathways were not directly segmented.

### 2.7 Statistical analyses

Previous studies by our group have reported on associations between anthropometric and SES measures (Jensen et al., 2019a). We also examined this relationship in this cohort of six year-olds by testing for correlations between anthropometric measures and maternal education and (log of) income-to-needs.

To address our first line of inquiry, that diminished growth predicts brain morphometry, we submitted total and regional volumetric and surface-based measures to semipartial correlation analyses controlling for sex and age at time of scan. As measures were predominantly distributed normally, according to to the D'Agostino & Pearson omnibus normality test, parametric testing was implemented for all correlation analyses. discovery rate (FDR) corrections for multiple comparisons were applied separately for volumetric, surface area, cortical thickness, sulcal depth, and mean curvature and for total and regional analyses (Benjamini and Hochberg, 1995). However, tests with HAZ, WAZ, and WHZ were corrected for multiple comparisons altogether. Thresholds were set at p_FDR_ < 0.05. To address whether SES predicts brain morphometry, as hypothesized, this procedure was repeated replacing anthropometric measures with measures of maternal education and income-to-needs. All brain-anthropometry and brain-SES associations with global measures of volume and surface area were re-computed with head circumference entered as an additional covariate of no interest. All brain-anthropometry and brain-SES associations with regional measures of volume and surface area were also re-computed with total brain volume entered as an additional covariate of no interest.

We also hypothesized that diminished growth mediates relationships between measures of SES and brain morphometry. Although the predictor and mediator included in the mediation model were collected at similar developmental stages, with SES preceding anthropometry by approximately eight months, they reflect different time frames. Specifically, SES likely reflects children’s environments throughout most of their lives up until the time of measurement, whereas the anthropometric measures characterize diminished growth at the time of measurement. Indirect effects were examined whenever an anthropometric measure exhibited a significant (after FDR correction for multiple comparisons) association with measures of SES and brain morphometry. Indirect effects were reported as significant when the 95% confidence intervals (based on 10,000 bootstrapped samples) for their proportion of the total effect (indirect + direct effect = total effect) did not include 0. This process was repeated for our third line of inquiry: whether measures of brain morphometry mediated associations between anthropometric measures and general cognitive ability (i.e., FIQ).

Correlational tests were conducted in Matlab and indirect effects were examined using the Mediation package in R. Brain maps were generated using the ggseg() function in R. All code used for statistical analyses and visualizations is housed in an openly available repository at https://github.com/TeddyTuresky/BrainMorphometry_DiminishedGrowth_BEANstudy_2021.

## 3 Results

### 3.1 Socioeconomic status (SES) is associated with diminished growth

We first examined the association between anthropometric indicators of stunting (i.e., height-for-age, HAZ), underweight (i.e., weight-for-age, WAZ), and wasting (i.e., weight-for-height, WHZ) and measures associated with SES. Maternal education correlated positively with HAZ (r = 0.29, p = 0.0088) and WAZ (r = 0.27, p = 0.017), but not with WHZ (r = 0.20, p = 0.083). Income-to-needs, which was log-transformed due to its skew (Noble et al. 2015), was associated with HAZ (r = 0.27, p = 0.017), WAZ (r = 0.30, p = 0.0075), and WHZ (r = 0.26, p = 0.021). All were significant at p_FDR_ < 0.05 except for between maternal education and WHZ. In summary, we observed that measures of SES were associated with diminished growth.

### 3.2 Diminished growth predicts brain morphometry

We next examined associations between anthropometric indicators of diminished growth and measures of brain structure, including global and regional volumetric and surface-based measures. Correlations reported for main analyses have been corrected for multiple comparisons at p_FDR_ < 0.05 except where otherwise noted, and uncorrected p-values are reported for additional information.

#### 3.2.1 Global measures

**Fig. 3:**
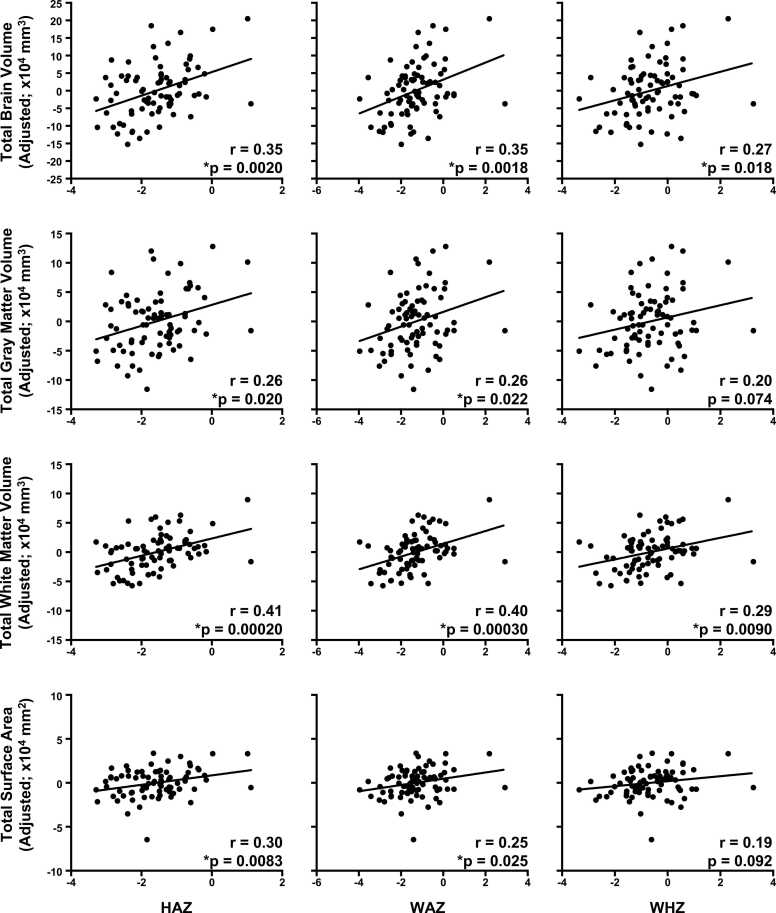
Diminished growth measured at two years predicts global measures of brain morphometry. Relationships were computed using semipartial correlations with brain measures adjusted for age and sex. Strongest effects were observed for total white matter volume and for HAZ. Please note, FDR correction for multiple comparisons were performed separately for volumetric and surface-based measures. N = 79. *p_FDR_ < 0.05.

Global measures of brain morphometry are summarized in Supplementary Table 1. For volumetric measures, total brain volume (TBV), total gray matter volume (GMV), and total white matter volume (WMV) correlated positively with HAZ, WAZ, and WHZ (Fig. 3). Total surface area (SA) across all cortical regions was associated with HAZ and WAZ, but not WHZ. Average sulcal depth also correlated positively associated with HAZ (r = 0.28, p = 0.012), but not WAZ (r = 0.23, p = 0.040) or WHZ (r = 0.17, p = 0.013) after FDR correction. Neither average cortical thickness nor average mean curvature was associated with any anthropometric measures (p > 0.05). When accounting for head circumference, the only associations to remain significant were between WMV and HAZ (r = 0.29, p < 0.05) and WAZ (r = 0.26, p < 0.05).

#### 3.2.2 Regional measures

**Fig. 4:**
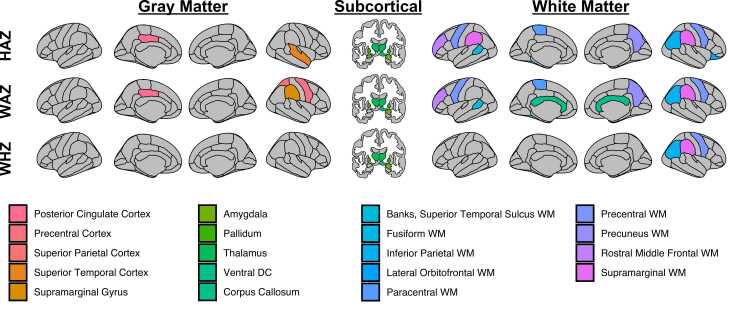
Diminished growth predicts regional brain volume. Associations occurred mostly in subcortical and white matter regions and for HAZ and WAZ. All brain maps have been p < 0.05 FDR-corrected for multiple comparisons for each anthropometric indicator. Please note that cerebellum white matter and unsegmented white matter are not depicted (please see Supplementary Table 2). WM, white matter; DC, diencephalon.

To investigate whether global measures of volume, surface area, and sulcal depth were driven by specific areas of the brain, we also examined brain-anthropometric associations in individual structures parcellated by Mindboggle. Volumetric measurescorrelated positively with HAZ and WAZ mainly in subcortical gray matter and white matter regions, and with WHZ only in white matter regions (Fig. 4; Supplementary Table 2). Sulcal depth in right caudal anterior cingulate was associated with HAZ (r = 0.39; p = 0.00044). However, no other surface-based measures were associated with diminished growth after FDR correction. When accounting for TBV, the number of significant regional brain-anthropometry associations were limited to left pallidum (r = 0.24, p < 0.05) and white matter volumes (r > 0.22, p < 0.05). Overall, diminished growth predicted brain morphometry, and these associations were most robust between HAZ and WAZ and brain volume in subcortical and white matter regions.

### 3.3 SES relates to brain morphometry

We next examined whether global and regional volumetric and surface-based measures were associated with measures of SES. As in the previous subsection, correlations reported for main analyses have been corrected at p_FDR_ < 0.05, and uncorrected p-values are reported for additional information.

#### 3.3.1 Global measures

**Fig. 5:**
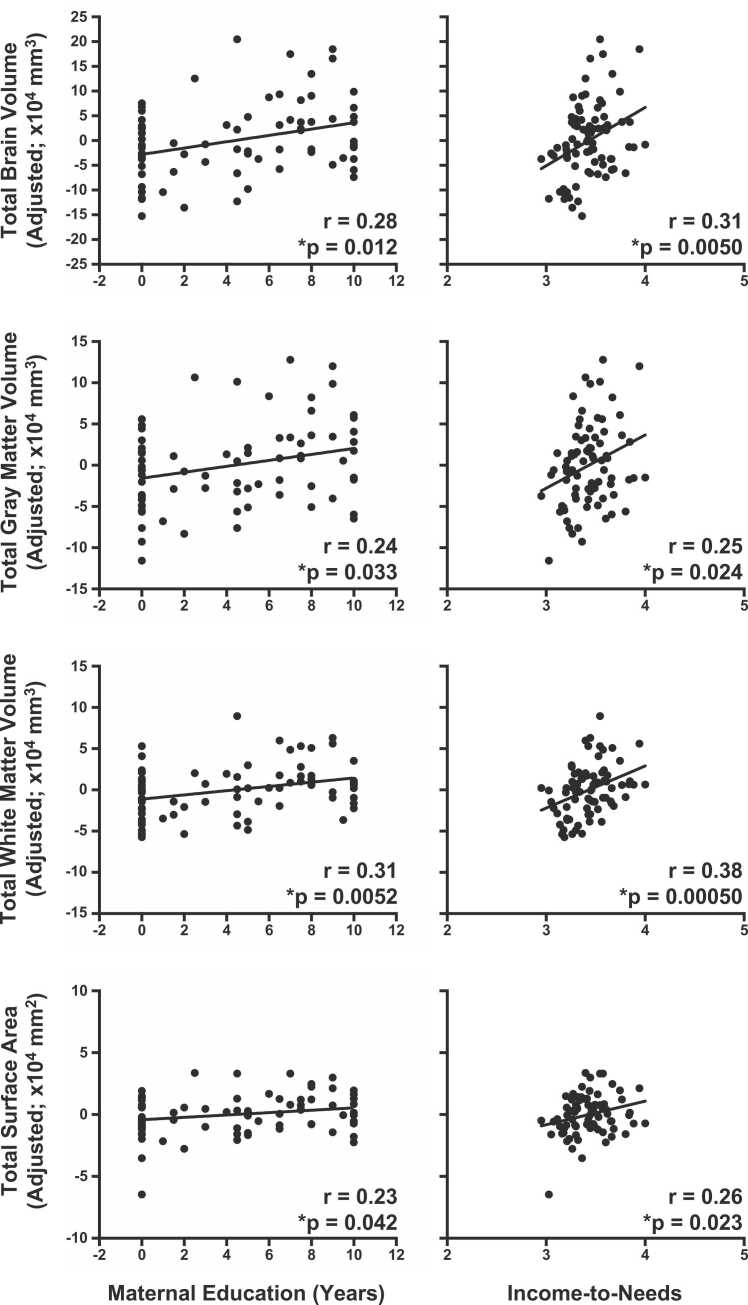
Childhood SES as measured with maternal education and income-to-needs relates to global measures of brain morphometry. Relationships were computed using semipartial correlations with brain measures adjusted for age and sex. Income-to-needs was computed as the log of household monthly income in Bangladeshi Taka divided by number of household members. Strongest effects were observed for total white matter volume. Please note, FDR correction for multiple comparisons were performed separately for volumetric and surface-based measures. N = 79. *p_FDR_ < 0.05.

In terms of global measures, TBV, GMV, WMV, and SA correlated positively with maternal education and income-to-needs (Fig. 5), which was consistent with brain-anthropometry findings. No surface-based measures apart from SA were associated with either maternal education or income-to-needs. When accounting for head circumference, only SES associations with WMV remained significant (maternal education: r = 0.34, p < 0.05; income-to-needs: r = 0.31, p < 0.05).

#### 3.3.2 Regional measures

Subsequently, regional associations between measures of SES and brain morphometry were examined. As with anthropometric measures, volumetric measures correlated positively with maternal education and, to a lesser extent, income-to-needs mainly in subcortical gray matter and white matter regions (Supplementary Table 3). Nevertheless, the only brain regions exhibiting brain-SES and brain-anthropometry associations were left pallidum, right ventral diencephalon, and bilateral unsegmented white matter. The only regional surface-based measure associated with SES was surface area, with associations between maternal education and right pars triangularis (r = 0.35; p = 0.0016) and between maternal education and right rostral middle frontal cortex (r = 0.36; p = 0.00097). Most regional volumetric associations with SES remained significant after accounting for TBV (r > 0.22, p < 0.05), as did the association between maternal education and right pars triangularis surface area (r = 0.25, p < 0.05).

### 3.4 Diminished growth mediates the relationship between SES and brain morphometry

We next examined indirect pathways between SES and brain morphometry via diminished growth. As a precondition for mediation, the mediator (i.e., diminished growth) must be associated with both the predictor (i.e., measure of SES) and outcome (i.e., brain morphometry). Thus, we examined indirect effects only where HAZ, WAZ, or WHZ related to maternal education or income-to-needs and volumetric or surface-based measures. Finally, indirect effects were reported when the 95% confidence intervals (CI), based on 10,000 bootstrapped samples, for their proportion of the total effect did not include 0 (please see Methods).

#### 3.4.1 Global measures

For global volumetric measures, HAZ mediated the relationship between maternal education and TBV (proportion mediated = 0.32, CI [0.044 0.89], p = 0.018) and TWM (proportion mediated = 0.36, CI [0.074 1.04], p = 0.0094) and between income-to-needs and TBV (proportion mediated = 0.29, CI [0.046 0.81], p = 0.015) and TWM (proportion mediated = 0.29, CI [0.067 0.68], p = 0.0064). WAZ also partially mediated the association between maternal education and TBV (proportion mediated = 0.27, CI [0.015 0.82], p = 0.034) and TWM (proportion mediated = 0.31, CI [0.034 0.94], p = 0.021) and between income-to-needs and TBV (proportion mediated = 0.28, CI [0.012 0.78], p = 0.040) and TWM (proportion mediated = 0.30, CI [0.048 0.70], p = 0.015). No indirect effects were observed for WHZ, and associations between SES and surface-based brain measures were not mediated by diminished growth.

#### 3.4.2 Regional measures

**Fig. 6:**
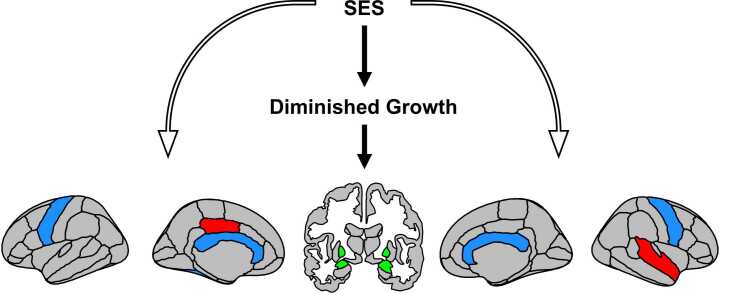
Diminished growth mediates the relationship between socioeconomic status (SES) and regional brain volume. Brain maps depict all brain regions whose association with maternal education or income-to-needs were mediated by HAZ, WAZ, or WHZ. Indirect effects (filled arrows) were mainly in subcortical gray matter (green) and white matter (blue), with some exceptions in cortical gray matter (red). Direct pathways (unfilled arrows) are also shown to signify that diminished growth mediates a proportion, but not all, of the association between SES and brain volume. Please note that cerebellum white matter and unsegmented white matter are not depicted. (For interpretation of the references to color in this figure legend, the reader is referred to the web version of this article.).

We next examined indirect effects with regional brain measures. Unlike with global brain measures, which correlated with both predictor and mediator variables, very few regional measures were associated with both predictors and mediators (left pallidum, right ventral diencephalon, and bilateral unsegmented white matter). However, modern models of mediation do not stipulate an association between predictor and outcome (Hayes, 2009). Therefore, we examined indirect pathways for any brain region related to an anthropometric measure. As with associations between diminished growth and brain volume, indirect effects were observed mainly in subcortical and white matter regions (Fig. 6). Notably, relatively consistent mediations (across several anthropometric and SES measure pathways; please see Supplementary Table 4) were observed for right amygdala, corpus callosum, and bilateral unsegmented white matter. In summary, diminished growth mediated the relation between maternal education and income-to-needs and global and regional (mainly subcortical and white matter) volumes.

### 3.5 Brain morphometry mediates the association between diminished growth and IQ

We next examined relationships between anthropometric measures and full-scale IQ (FIQ) scores. After FDR correction for multiple comparisons (p_FDR_ < 0.05), FIQ correlated positively with HAZ (r = 0.35, p = 0.0016), WAZ (r = 0.37, p = 0.00080), and WHZ (r = 0.33, p = 0.0034). Global measures of brain morphometry were also related to FIQ, namely TBV (r = 0.24, p = 0.032), GMV (r = 0.25, p = 0.024), average cortical thickness (r = 0.26, p = 0.022), and average sulcal depth (r = 0.25, p = 0.026), as were regional volumes in left pallidum (r = 0.45, p = 0.000027), and right ventral diencephalon (r = 0.42, p = 0.00010).

**Fig. 7:**
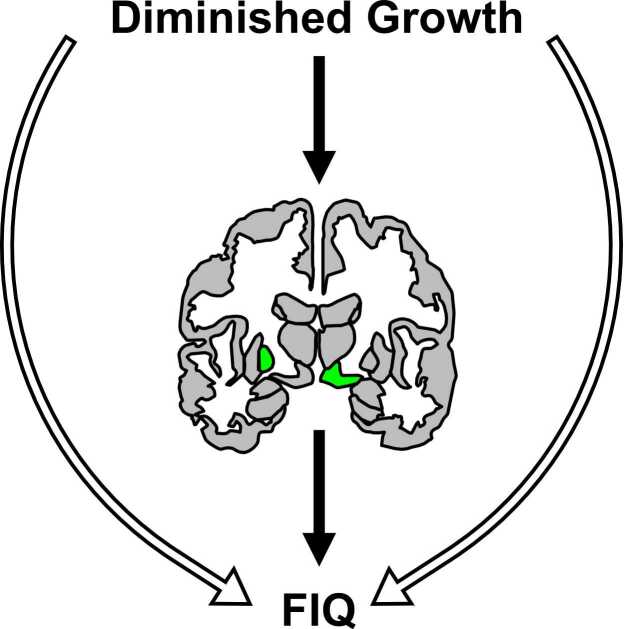
Brain volumes in left pallidum and right ventral diencephalon partially mediate the association between diminished growth and FIQ. Diminished growth includes HAZ and WAZ. Indirect pathways (filled arrows) show diminished growth to FIQ via brain volume and direct pathways (unfilled arrows) signify that left pallidum and right ventral diencephalon volumes mediate a proportion, but not the entirety, of the association between diminished growth and IQ.

Finally, we tested indirect effects between diminished growth and FIQ via global and regional measures of brain morphometry for the ten brain estimates correlated with both anthropometric measures and FIQ. Of these, mediation effects were observed for the pathways between HAZ and FIQ via left pallidum (proportion mediated = 0.39, CI [0.11 0.99], p = 0.0034) and right ventral diencephalon (proportion mediated = 0.30, CI [0.050 0.87], p = 0.013) and between WAZ and FIQ via left pallidum (proportion mediated = 0.35, CI [0.10 0.86], p = 0.0032) and right ventral diencephalon (proportion mediated = 0.29, CI [0.046 0.79], p = 0.015; Fig. 7). In summary, we observed links between diminished growth and FIQ and these associations were partially mediated by left pallidum and right ventral diencephalon volumes.

## 4 Discussion

Malnutrition occurs frequently in children growing up in extreme poverty and is thought to substantially derail typical cognitive development (Jensen et al., 2017). In this study, we observed that forms of diminished growth—i.e., stunting, underweight, and wasting—which are proxies for malnutrition (Black et al., 2013, de Onis and Branca, 2016, Schnee et al., 2018, Stewart et al., 2013), predicted brain morphometry and these effects were strongest in subcortical and white matter regions. Further, diminished growth mediated links between measures of childhood SES and brain volume. Finally, left pallidum and right ventral diencephalon volumes partially mediated associations between diminished growth and full-scale IQ (FIQ).

Diminished growth at roughly two years predicted global and regional morphometric measures at six years, including total brain volume, total gray matter volume, total white matter volume, and total surface area. Excepting surface area, which exhibited associations with height-for-age (HAZ) and weight-for-age (WAZ), the findings overall do not support robust relationships between surface-based measures and diminished growth. Overall, results for total brain volume are consistent with clinical findings of cerebral atrophy in young children suffering from wasting and acute malnutrition (Gunston et al., 1992, Hazin et al., 2007, Atalabi et al., 2010, El-Sherif et al., 2012, Kumar et al., 2016). The particularly strong predictions of global white matter volume from diminished growth support earlier work by our group showing that stunting and underweight inversely relate to total white, but not total gray, matter volume in infancy (Turesky et al., 2020). Results are also consistent with clinical findings of gross white matter atypicalities in children suffering from wasting and acute protein energy malnutrition (Gunston et al., 1992, Hazin et al., 2007, Atalabi et al., 2010, El-Sherif et al., 2012, Kumar et al., 2016) and inflammation (Yoon et al., 1997, Duggan et al., 2001, Hansen-Pupp et al., 2005), which is strongly linked with malnutrition (Rytter et al., 2014; for a discussion, please see John et al., 2017). The relationship between inflammation and white matter also has a basis in animal work, with inflammatory cytokines disrupting oligodendrocyte maturation and consequently reducing myelination (Favrais et al., 2011). Taken altogether, we add to the literature with quantitative estimates of brain volume and with topographical maps that depict where diminished growth predicts gray and, particularly, white matter volume.

With regard to subcortical structures, findings in the ventral diencephalon, which includes the hypothalamus (Makris et al., 2008), were also compelling because this area is thought to be a nexus for complex brain-gut-inflammation interactions (Keunen et al., 2015). Specifically, we identified an association between brain volume and a measure that is thought to reflect malnutrition (to which inflammation is linked (Rytter et al., 2014)) in an area with bidirectional connections with the gut (De Palma et al., 2014) and with a role in inflammatory regulation via the hypothalamic-pituitary-adrenal (HPA) axis (Irwin and Cole, 2011). Moreover, the gut microbiome is thought to play a vital role in the development of the HPA axis (Sudo, 2014). That ventral diencephalon was shown to mediate the relationship between diminished growth and FIQ further supports this, as well as the hypothesis that this area affects cognitive function (Lupien et al., 2009). Left pallidum was also shown to mediate the relationship between diminished growth and FIQ, and this area may affect cognition through known anatomical projections to prefrontal cortex, as shown in non-human primates (Middleton and Strick, 1994). Importantly, if diminished growth accurately represents malnutrition, then one might expect it to relate to measurements in all brain areas. However, brain regions’ susceptibility to nutrient deficits depends on the timing and degree of the deficit and that region’s need for the nutrient at that particular time during development (Prado and Dewey, 2014, Cusick and Georgieff, 2016). That these two factors vary considerably across development (e.g., brain regions develop at different rates) provides a strong basis for regional variation in associations between diminished growth and anthropometric measures. Nevertheless, our findings, particularly those corresponding to specific brain regions, should be interpreted with caution until future studies using direct, biological measures of malnutrition and confirmatory statistical techniques may be brought to bear on brain morphometry.

Our work also addresses a persistent gap in neuroscientific research on SES, namely that the overwhelming majority of such research has been conducted in high-resource settings. Our findings in children growing up in a low-resource setting support studies in high-resource countries reporting associations between socioeconomic status (SES) and total brain volume (McDermott et al., 2019), gray matter volume (Luby et al., 2013, Mackey et al., 2015, McDermott et al., 2019), white matter volume (Luby et al., 2013, McDermott et al., 2019), and surface area (Noble et al., 2015, McDermott et al., 2019). Regional effects were observed in the striatum, which was also found previously (McDermott et al., 2019). The most notable departures from studies in high-resource settings were the absences of effects for cortical thickness (Mackey et al., 2015, Noble et al., 2015, McDermott et al., 2019) and for the hippocampus (Noble et al., 2012, Noble et al., 2015, Hair et al., 2015, McDermott et al., 2019), suggesting that the brain correlates of SES may differ between low- and high-resource settings. Additionally, by comparison with reports of age-comparable, typically developing children growing up in the U.S., the cohort in Bangladesh exhibited lower total brain volume (Lenroot et al., 2007) and total gray and white matter volumes (Giedd et al., 1999, Sanchez et al., 2012). Future cross-cultural studies will need to directly compare brain volumes processed using identical methods to test this.

There are many pathways through which SES disadvantage may alter brain development (Raizada and Kishiyama, 2010, Gianaros and Hackman, 2013, Johnson et al., 2016, Jensen et al., 2017, Lipina and Evers, 2017, Farah, 2018). However, very few intermediary risk factors along these pathways have been tested in formal mediation models, which are necessary (though insufficient) for establishing causal pathways (for a discussion, please see Farah, 2017). As hypothesized, we found that diminished growth mediated the relationship between SES and global and regional brain volume. It is noteworthy that links between both measures of SES—maternal education and income-to-needs—and right amygdala were mediated by two of the three measures of diminished growth—HAZ and WAZ. This region’s structure and function exhibited associations with SES in some (Luby et al., 2013, McDermott et al., 2019, Turesky et al., 2019), but not other SES studies (Hanson et al., 2011, Jednorog et al., 2012, Noble et al., 2015), and it was proposed that the presence of an SES-amygdala association may depend on the degree of SES disadvantage experienced by the study cohort (Brito and Noble, 2014), which is consistent with our findings in children growing up in extreme poverty.

The links of SES to diminished growth and to brain morphometry via direct and indirect pathways merits discussion in the context of our earlier work, in which we did not observe these links. Unlike the present study, the prior work was conducted in children at infancy, before long-term exposure to poverty (Turesky et al., 2020), suggesting that the effects of poverty take time to accumulate (Nelson, 2017). This explanation also makes sense when considering that the association between diminished growth and poverty is age-dependent (Victora et al., 2010), with SES linked with anthropometric measures at three years of age, but not in infancy (Jensen et al., 2019a). This age-dependence and/or accumulated exposure to poverty may also explain why we did not observe relations between gray matter and diminished growth in infancy (Turesky et al., 2020), but we did here, in older children. Overall, measures of SES have widespread and substantial associations with subcortical and white matter volumes and these associations are partially mediated by diminished growth (as a proxy of malnutrition).

The present work had four notable limitations. The first is that diminished growth is a *proxy* for malnutrition (Bhutta et al., 2017a, Black et al., 2013, de Onis and Branca, 2016, Schnee et al., 2018), rather than a direct measure. However, malnutrition is a complex factor, and using specific measures of nutrient deficiencies (e.g., zinc or iron) may not have adequately captured the full impact of myriad deficiencies on human development. The second limitation is that HAZ, WAZ, and WHZ (i.e., weight-for-height) were all examined as potential mediators. While each were involved in significant indirect pathways separately, their independent contributions as mediators is complicated by collinearity between them (e.g., HAZ and WAZ: r = 0.87; p = 3.4 × 10^-25^). For this reason, indirect pathways linking SES to brain morphometry are characterized as mediated by diminished growth in general. The third limitation is that the measures for diminished growth and SES were not contemporaneous with MRI acquisition. However, while it is possible that these measures would change by the time the child reaches six years of age, at least across measurements, these estimates were relatively constant (please see Methods for details). The final limitation is a reminder that while the models demonstrating how SES could affect brain development presume causal links (Noble et al., 2012), the design of this study tested correlations only. Findings should be viewed cautiously in light of these limitations.

Finally, the vast majority of databases comprise data from white U.S. and European individuals (Cell Editorial Team, 2020). In contrast, the children in this study grew up in an impoverished area of Bangladesh, making them an extremely underrepresented population in neuroscientific research, both racially and socioeconomically. As such, involving them in MRI studies such as the present one is critical to begin to address inequity in developmental cognitive neuroscience research. We encourage others to chart similar paths in their own lines of inquiry.

## 5 Conclusions

This study comprehensively examines links among brain morphometry, diminished growth, SES, and general cognitive ability in children growing up in a low-resource setting in Bangladesh. Our findings show that diminished growth, a proxy for malnutrition, predicts brain morphometry and mediates associations between SES and brain morphometry. In doing so, this study suggests that malnutrition may be one pathway through which poverty alters brain development; however, as malnutrition was not directly measured, this conclusion must be viewed with caution. Although future longitudinal studies with nutritional interventions will be needed to test the causality of this pathway, this study has important implications for the role of nutrition in children growing up in low-resource settings.

## Declaration of Competing Interest

The authors declare that they have no known competing financial interests or personal relationships that could have appeared to influence the work reported in this paper.

## Data Availability

Data and code have been made openly available at https://openneuro.org/datasets/ds003877/versions/1.0.1 (data) and https://github.com/TeddyTuresky/BrainMorphometry_DiminishedGrowth_BEANstudy_2021 (code).
