# Supplementary material for: Brain morphometry and diminished physical growth in Bangladeshi children growing up in extreme poverty: A longitudinal study
Source: Dev Cogn Neurosci. 2021 Oct 26;52:101029. doi: 10.1016/j.dcn.2021.101029 (PMC8605388; doi:10.1016/j.dcn.2021.101029)
Supplement: Supplementary file 1 — Supplementary material [file mmc1.docx]

**SUPPLEMENTARY TABLES**

| **Supplementary Table 1. Global Estimates of Brain Morphometry** | | |
| --- | --- | --- |
| Total brain volume (mm^3^) | 1087300 (82428) |  |
| Total gray matter volume (mm^3^) | 685850 (55271) |  |
| Total white matter volume (mm^3^) | 374940 (32442) |  |
| Total surface area (mm^2^) | 199000 (17371) |  |
| Average Cortical Thickness (mm) | 2.9092 (0.0995) |  |
| Average Sulcal Depth (mm) | 5.9399 (0.3024) |  |
| Average Curvature (mm) | -3.6173 (0.4230) |  |

| **Supplementary Table 2. Brain-Anthropometry Relationships** | |  |
| --- | --- | --- |
| ***HAZ*** | **r** | **p_unc_ (x 10^-3^)** |
| L. Posterior Cingulate Cortex | 0.33 | 2.7 |
| R. Superior Temporal Cortex | 0.34 | 2.0 |
| L. Amygdala | 0.33 | 3.4 |
| L. Pallidum* | 0.41 | 0.19 |
| L. Thalamus | 0.36 | 1.0 |
| R. Amygdala | 0.40 | 0.28 |
| R. Pallidum | 0.38 | 0.47 |
| R. Thalamus | 0.31 | 5.2 |
| R. Ventral Diencephalon | 0.33 | 2.6 |
| L. Banks, Superior Temporal Sulcus White Matter | 0.30 | 6.8 |
| L. Cerebellum White Matter | 0.35 | 1.6 |
| L. Fusiform White Matter* | 0.40 | 0.22 |
| L. Paracentral White Matter* | 0.39 | 0.42 |
| L. Precentral White Matter | 0.32 | 4.0 |
| L. Rostral Middle Frontal White Matter | 0.34 | 2.2 |
| L. Supramarginal White Matter | 0.33 | 2.7 |
| L. Unsegmented White Matter | 0.36 | 1.1 |
| R. Inferior Parietal White Matter* | 0.42 | 0.14 |
| R. Lateral Orbitofrontal White Matter | 0.32 | 4.6 |
| R. Precentral White Matter | 0.36 | 1.0 |
| R. Precuneus White Matter | 0.36 | 1.2 |
| R. Supramarginal White Matter | 0.31 | 5.3 |
| R. Unsegmented White Matter* | 0.42 | 0.11 |
|  |  |  |
| ***WAZ*** | **r** | **p_unc_ (x 10^-3^)** |
| L. Posterior Cingulate Cortex | 0.34 | 2.1 |
| R. Precentral Cortex | 0.31 | 6.0 |
| R. Superior Parietal Cortex | 0.30 | 6.3 |
| R. Supramarginal Cortex | 0.32 | 3.7 |
| L. Pallidum* | 0.39 | 0.33 |
| L. Thalamus | 0.35 | 1.8 |
| R. Amygdala | 0.33 | 2.7 |
| R. Pallidum | 0.35 | 1.8 |
| R. Thalamus | 0.32 | 4.7 |
| R. Ventral Diencephalon | 0.34 | 2.0 |
| L. Banks, Superior Temporal Sulcus White Matter* | 0.33 | 2.9 |
| L. Cerebellum White Matter* | 0.36 | 1.1 |
| L. Fusiform White Matter | 0.34 | 2.1 |
| L. Paracentral White Matter* | 0.38 | 0.55 |
| L. Precentral White Matter | 0.30 | 6.8 |
| L. Rostral Middle Frontal White Matter | 0.30 | 6.4 |
| L. Unsegmented White Matter* | 0.40 | 0.24 |
| R. Inferior Parietal White Matter* | 0.42 | 0.11 |
| R. Precentral White Matter* | 0.43 | 0.063 |
| R. Precuneus White Matter | 0.33 | 3.0 |
| R. Supramarginal White Matter* | 0.38 | 0.56 |
| R. Unsegmented White Matter* | 0.45 | 0.037 |
| Corpus Callosum | 0.30 | 6.4 |
|  |  |  |
| ***WHZ*** | **r** | **p_unc_ (x 10^-3^)** |
| L. Unsegmented White Matter* | 0.36 | 1.1 |
| R. Inferior Parietal White Matter* | 0.37 | 0.90 |
| R. Precentral White Matter* | 0.43 | 0.072 |
| R. Supramarginal White Matter* | 0.37 | 0.68 |
| R. Unsegmented White Matter* | 0.39 | 0.41 |
| *p < 0.05 when controlling for total brain volume |  |  |

| **Supplementary Table 3. Brain-SES Relationships** | |  |
| --- | --- | --- |
| ***Maternal Education*** | **r** | **p_unc_ (x 10^-3^)** |
| R. Pars Triangularis* | 0.37 | 0.87 |
| R. Rostral Middle Frontal Cortex* | 0.39 | 0.32 |
| R. Superior Frontal Cortex | 0.34 | 2.1 |
| L. Caudate | 0.32 | 4.0 |
| L. Pallidum* | 0.37 | 0.91 |
| L. Putamen* | 0.37 | 0.91 |
| L. Ventral Diencephalon | 0.34 | 2.1 |
| R. Putamen* | 0.41 | 0.20 |
| R. Ventral Diencephalon* | 0.39 | 0.44 |
| L. Unsegmented White Matter | 0.36 | 1.3 |
| R. Caudal Anterior Cingulate White Matter* | 0.40 | 0.27 |
| R. Insula White Matter | 0.36 | 1.0 |
| R. Pars Triangularis White Matter* | 0.36 | 1.2 |
| R. Rostral Middle Frontal White Matter* | 0.37 | 0.68 |
| R. Superior Frontal White Matter* | 0.36 | 1.0 |
| R. Unsegmented White Matter* | 0.39 | 0.44 |
|  |  |  |
| ***Income-to-Needs*** | **r** | **p_unc_ (x 10^-3^)** |
| L. Pallidum | 0.36 | 1.1 |
| L. Ventral Diencephalon | 0.38 | 0.48 |
| R. Ventral Diencephalon* | 0.41 | 0.15 |
| L. Lateral Orbitofrontal White Matter | 0.39 | 0.43 |
| L. Parahippocampal White Matter | 0.34 | 2.1 |
| L. Unsegmented White Matter | 0.35 | 1.5 |
| R. Cerebellum White Matter* | 0.38 | 0.50 |
| R. Unsegmented White Matter* | 0.41 | 0.20 |
| *p < 0.05 when controlling for total brain volume |  |  |

| **Supplementary Table 4. Indirect Effects** |  |  |  |  |
| --- | --- | --- | --- | --- |
| *Maternal Education → HAZ → Brain Volume* | Estimate | Lower CI | Upper CI | p |
| R. Superior Temporal Cortex | 0.28 | 0.027 | 0.95 | 0.027 |
| L. Amygdala | 0.30 | 0.053 | 0.90 | 0.020 |
| L. Pallidum | 0.28 | 0.065 | 0.92 | 0.0098 |
| R. Amygdala | 0.38 | 0.11 | 1.0 | 0.0092 |
| R. Pallidum | 0.38 | 0.067 | 1.5 | 0.021 |
| L. Cerebellum White Matter | 0.37 | 0.067 | 1.5 | 0.017 |
| L. Unsegmented White Matter | 0.25 | 0.031 | 0.80 | 0.022 |
| R. Unsegmented White Matter | 0.28 | 0.046 | 0.82 | 0.011 |
|  |  |  |  |  |
| *Maternal Education → WAZ → Brain Volume* | Estimate | Lower CI | Upper CI | p |
| L. Pallidum | 0.26 | 0.043 | 0.88 | 0.017 |
| R. Amygdala | 0.28 | 0.033 | 0.98 | 0.028 |
| R. Pallidum | 0.31 | 0.016 | 1.4 | 0.038 |
| Corpus Callosum | 0.28 | 0.022 | 1.6 | 0.035 |
| L. Cerebellum White Matter | 0.36 | 0.060 | 1.5 | 0.020 |
| L. Unsegmented White Matter | 0.27 | 0.045 | 0.89 | 0.015 |
| R. Unsegmented White Matter | 0.28 | 0.045 | 0.85 | 0.013 |
|  |  |  |  |  |
| *Income-to-Needs → HAZ → Brain Volume* | Estimate | Lower CI | Upper CI | p |
| R. Superior Temporal Cortex | 0.33 | 0.070 | 0.98 | 0.0080 |
| L. Thalamus | 0.17 | -0.013 | 0.57 | 0.074 |
| R. Amygdala | 0.26 | 0.015 | 1.5 | 0.040 |
| L. Precentral White Matter | 0.24 | 0.045 | 0.64 | 0.012 |
| R. Precentral White Matter | 0.32 | 0.045 | 0.96 | 0.019 |
| R. Unsegmented White Matter | 0.24 | 0.054 | 0.55 | 0.0054 |
|  |  |  |  |  |
| *Income-to-Needs → WAZ → Brain Volume* | Estimate | Lower CI | Upper CI | p |
| L. Posterior Cingulate Cortex | 0.29 | 0.068 | 0.81 | 0.0054 |
| R. Amygdala | 0.30 | 0.034 | 1.6 | 0.031 |
| L. Cerebellum White Matter | 0.32 | 0.036 | 1.0 | 0.020 |
| L. Fusiform White Matter | 0.28 | 0.077 | 0.71 | 0.0052 |
| L. Precentral White Matter | 0.38 | 0.023 | 1.6 | 0.036 |
| L. Unsegmented White Matter | 0.31 | 0.074 | 0.74 | 0.0066 |
| R. Unsegmented White Matter | 0.29 | 0.066 | 0.64 | 0.0082 |
|  |  |  |  |  |
| *Income-to-Needs → WHZ → Brain Volume* | Estimate | Lower CI | Upper CI | p |
| L. Unsegmented White Matter | 0.23 | 0.021 | 0.62 | 0.026 |
| R. Unsegmented White Matter | 0.21 | 0.0071 | 0.52 | 0.041 |
